# Transmutations within the Streptococcus-Pneumococcus Group

**Published:** 1914-05

**Authors:** 


TRANSMUTATIONS WITHIN THE STREPTOCOCCUS-PNEUMOCOCCUS GROUP.—Rosenow reports the transmutation of streptococci into pneumococci and vice versa. In twenty-four instances strains of streptococci have been converted into pneumococci. Thirteen strains of pneumococci were made to correspond to hemolytic streptococci; transformation being complete as shown by every known test. It seems probable that transformations take place in foci of infection where streptococci and pneumococci possibly evolve new affinities for tissues.

